# Knowledge, Attitudes and Practices Regarding Pre-Exposure Prophylaxis (PrEP) in a Sample of Italian Men Who Have SEX with MEN (MSM)

**DOI:** 10.3390/ijerph18094772

**Published:** 2021-04-29

**Authors:** Gianluca Voglino, Maria Rosaria Gualano, Stefano Rousset, Pietro Forghieri, Isabella Fraire, Fabrizio Bert, Roberta Siliquini

**Affiliations:** 1 Department of Public Health Sciences and Paediatrics, University of Turin, 10126 Turin, Italy; gianluca.voglino@unito.it (G.V.); stefano.rousset@unito.it (S.R.); pietro.forghieri@unito.it (P.F.); isabella.fraire@edu.unito.it (I.F.); fabrizio.bert@unito.it (F.B.); roberta.siliquini@unito.it (R.S.); 2 AOU City of Health and Science of Turin, Corso Bramante 88, 10126 Turin, Italy

**Keywords:** PrEP, MSM, HIV prevention, knowledge, sexual behavior

## Abstract

Background: Pre-exposure prophylaxis (PrEP) is suitable for high human immunodeficiency virus (HIV)-infection risk people, foremost among whom are males who have sex with other males (MSM). This study evaluated knowledge, attitudes and practices regarding PrEP in a sample of Italian MSM, in order to hypothesize strategies to implement PrEP awareness and use. No previous study has assessed this issue; Methods: An online survey was given to an opportunistic sample of Italian MSM. The questionnaire investigated sexual behaviour and habits, HIV/acquired immune deficiency syndrome (AIDS) knowledge and PrEP awareness, attitudes and practices. Univariable and multivariable logistic regressions were conducted to identify factors associated with PrEP knowledge; Results: A total of 196 MSM participated in this survey. Overall data showed that 87.2% of participants knew what PrEP is, but only 7.5% have ever used it. The main reason for not using PrEP was the cost of the therapy (26.9%). The principal source of PrEP information was the Internet (68.4%). Being regularly tested for HIV was significantly associated with PrEP knowledge (adjusted odds ratio (AdjOR) = 3.16; confidence interval (CI) = 1.06–9.29); Conclusions: Knowledge regarding PrEP was well established, but PrEP use was not equally widespread. It is necessary to improve research on PrEP usage in order to PrEP access to be granted.

## 1. Introduction

The incidence rate of human immunodeficiency virus (HIV) infection in Italy has not changed since 2015 (5.7 new cases per 100,000 persons) similar to the European mean (5.8 new cases per 100,000 persons). The events are foremost due to sexual transmission. However, the numbers in the MSM (men who have sex with men) population greatly increased (31.8% in 2010 and 38.5% in 2017) [1]. The MSM population is, therefore, at high risk of HIV contagion, and adequate campaigns of information and prevention are needed.

Among the preventive instruments available, pre-exposure prophylaxis (PrEP) is gaining consensus in the scientific community. The prophylaxis is an association of two different antiretroviral drugs (tenofovir/emtricitabine). It has to be taken immediately before a high-HIV-risk sexual act or behaviour, or on a daily basis. Hence, PrEP requires a high level of adherence over time in order to be effective. According to a recent systematic review, PrEP adherence is high among MSM in high-income countries [2]. Thus, access to PrEP, rather than adherence, seems to be the strongest obstacle to PrEP use.

According to the most recent Italian guidelines, PrEP is suitable for MSM with at least one of the following conditions: at least one anal intercourse without condom use with an occasional partner positive for HIV or with an unknown serology; treatment of a sexual transmitted disease; previous PEP (post-exposure prophylaxis); chemsex (sexual act associated with psychotropic substances) [3]. In Italy, at the moment, costs for PrEP are not covered by the National Health Service and anyone who wants to use it has to pay for it and needs a prescription from an infectious disease specialist [4]. These requirements represent a barrier for the diffusion of PrEP knowledge and use in the interested community.

A significant number of international studies have demonstrated that PrEP, if taken correctly, is safe and highly effective in preventing HIV transmission [5,6,7]. Daily PrEP reduced the risk of HIV transmission by 86% in the PROUD clinical trial [8], while the IPERGAY clinical trial showed the same risk reduction using the on-demand schedule [9]. For this reason, in 2012 the World Health Organization has recommended PrEP for seronegative people with a heterosexual seropositive partner, and in 2015 this recommendation has been extended to MSM and intravenous drug users [10]. In August 2020, the Italian Ministry of Health approved the new National Plan for Prevention 2020–2025, which specifically considers the implementation of PrEP use as a strategic objective for HIV prevention [11].

Knowledge and willingness to use PrEP among people at high risk of HIV infection were evaluated in a Spanish study during the 2017 Gay Pride. The study showed that MSM have a limited awareness about PrEP, but a strong willingness to gain more information and possibly use it. In particular, 64% of participants were aware of PrEP, but only 33% knew correctly what PrEP was [12]. Similar results were obtained by a study conducted in China [13] and confirmed by a recent systematic review with meta-analysis, including 23 studies from numerous countries [14].

Another study was conducted in Italy in order to evaluate knowledge, attitudes and practices regarding PrEP and antiretroviral therapy in a sample of persons living with HIV/acquired immune deficiency syndrome (AIDS) patients. In this study, 45.6% of the patients stated that they were informed about PrEP; however, this result comes from a highly selected sample, informed about HIV and in contact with an infectious disease specialist [15]. Hence, this sample is not representative of the population which can obtain the highest benefit from PrEP use, namely HIV-negative subjects at high risk of contagion, such as MSM. To the best of our knowledge, no studies exists in our specific context regarding this population.

The main purpose of this study is, therefore, to evaluate knowledge, attitudes and practices regarding PrEP in a sample of Italian MSM, in order to hypothesize strategies for the implementation of PrEP awareness and use in this category of persons at high risk of HIV infection.

## 2. Materials and Methods

### 2.1. Study Design

The Health Belief Model (HBM) was used as theoretical method, as it contains several primary concepts that predict why people will take action to prevent, to screen for, or to control illness conditions [16]. A cross-sectional survey on an opportunistic sample of Italian MSM (men who have sex with men) was designed. The initial purpose was to select around 500 eligible participants from public spaces. However, due to the Covid-19 pandemic, to transform the collection process in an online survey was considered the best option. The questionnaires were distributed at a national level using the institutional social network account of the Department of Public Health Sciences (University of Torino) and through a snowball sampling.

Participants had to meet the following inclusion criteria: age ≥ 18 years, male gender, being able to understand the questionnaire and to sign an informed consent. Being exclusively attracted to females and being HIV-positive were exclusion criteria.

All the participants were adequately informed about the purposes of the study. Participation was voluntary and the researchers guaranteed the anonymity of the participants during data extraction and results analysis.

This study was approved by the Bioethical Committee of the University of Turin.

### 2.2. Questionnaire

After a literature search, a 38-item questionnaire was developed and divided in three sections. The researchers’ aim was to design a valid, reliable, clear, succinct questionnaire, considering the previous knowledge on the topic, the theoretical framework and their previous experience [17]. The questionnaire was distributed to participants in Italian and the variables were translated in English for publication purpose. All the items were written using multiple-choice questions.

In the first section, the socio-demographic characteristics of the sample were assessed (items 1–6).

In the second section, sexual behavior and habits were evaluated (items 7–22). These items were adapted from a published study [15] and from an online survey conducted in Spain in 2017 [12]. We added two items regarding the relational life of the subject (items 9–10), as the presence of different kinds of relation has been previously associated with risky behaviors [18]. Study’s term was extended from 6 to 12 months as described in literature [19]. Item 22 contains a list of questions investigating knowledge about HIV and prevention, adapted from a validated questionnaire present in literature [20] with the aim to assess perceived susceptibility and perceived severity, in line with the HBM.

In the third section, knowledge, attitudes and practices regarding PrEP were investigated (items 23–38). This part of the questionnaire was preceded by a brief definition of PrEP, in order to establish a common ground for each respondent in answering these questions, hence increasing results comparability. This section was built in order to investigate perceived benefits and perceived barriers, according to the HBM. Items were adapted from published studies [12,15] and targeted on PrEP use [8,21,22]. Items 35–38 also address self-efficacy coherently to the HBM.

### 2.3. Statistical Analysis

Descriptive analyses were performed for all variables and expressed as frequencies and percentage for categorical variables or median and interquartile range for continuous variables. In fact, normal distribution was assessed for continuous variables using the Shapiro–Wilk test. Differences between the groups defined by each outcome were investigated using chi-squared tests (when appropriate: Fisher’s exact test) and Mann–Whitney U tests (when appropriate: Kruskal–Wallis H test). Univariable and multivariable logistic regressions were conducted to assess the independent variables influence on the binary outcome (results expressed as odds ratio (OR), 95% confidence interval (CI)). The covariates included in multivariable models were selected using a stepwise backward selection process, with a univariable *p*-value ≤ 0.25 as the main criterion [23]. SPSS (v25) was used to perform analysis. A two-tailed *p*-value ≤ 0.05 was considered significant. Missing values were excluded.

## 3. Results

### 3.1. Description of the Sample

A total of 196 MSM participated in this survey. The median age was 31 years old. Socio-demographic characteristics and sexual behaviours of the participants are shown in Table 1. The vast majority (97.4%) was Italian and most of the participants (68.9%) had a University degree and worked as employees (35.2%). More than half of the respondents (54.6%) were single and 74.5% of them were sexually attracted exclusively to males. Less than half of the respondents were enrolled in a lesbian, gay, bisexual, and transgender (LGBT) association (38.3%) or in an anti-AIDS association (12.8%). Only 27% of the participants use regularly a condom with their stable partner, but 93.8% of them used it during occasional sex. According to the Italian guidelines for HIV-patients management [2], we defined six risky behaviours that increase the risk of HIV infection: having had more than one partner in the last 12 months, having had unprotected sexual intercourse in the last 12 months, having experimented with chemsex, having received money in exchange of sex, having used intravenous drugs, and having contracted a sexual-transmittable disease in the last 12 months. Median value of risky behaviours in our sample was 2.

### 3.2. Human Immunodeficiency Virus (HIV) Knowledge

Table 2 shows the 18 questions regarding HIV/AIDS knowledge. Our sample showed a very high level of knowledge, with a median of 17 and a percentage of 94.4% of correct answers.

### 3.3. Knowledge, Attitudes and Practices Regarding Pre-Exposure Prophylaxis (PrEP)

Most of the participants (91.1%) had heard of PrEP before, 87.2% of them knew what PrEP was, but only 7.5% of them had ever used it. More than half (68.4%) had talked about PrEP with friends or relatives, but only 34.5% with a health care worker. More than half of the participants stated that they would be more willing to use PrEP if they had more information about it (52.1%), if it were free (66.5%), or if it were purchasable without medical prescription (57.4%) (Table 3). Among the reasons for not using PrEP, the most significant were the high cost of the therapy (26.9%), fear of side effects (23.8%) and the belief of not being at risk for HIV (21.3%). Only 3.8% of the participants did not use PrEP because of the fear of being discriminated (Table 3). The principal sources of PrEP information were the Internet (68.4%) and friends, relatives and acquaintances (47.7%). Only 10.3% gained information from institutional channels, 7.5% from specialized physicians and just one participant (0.6%) from the general practitioner (Table 3).

### 3.4. Variables Associated with PrEP Knowledge

In the univariate analysis, the variables that showed the strongest association with PrEP knowledge were educational level higher than high school, being single, having had sex with more than one man in the last 12 months, being regularly tested for HIV, having received money in exchange for sex (*p* ≤ 0.25) (Table 4). These variables were further analysed in a multivariate logistic regression model. The results from the regression showed that being regularly tested for HIV is the strongest factor associated with PrEP knowledge (OR = 3.09; CI = 1.15–8.34), even when adjusting for the other variables included in the analysis (adjusted odds ratio (AdjOR) = 3.16; CI = 1.06–9.29). Other variables associated with PrEP knowledge were being single (OR = 2.96; CI = 1.14–6.01) and having had sex with more than one man in the last 12 months (OR = 3.94; CI = 1.48–6.89), but these results were not statistically significant when the model was adjusted for the other included variables (Table 5).

## 4. Discussion

The aim of this study was to evaluate knowledge, attitudes and practices regarding PrEP in a sample of Italian MSM.

In our sample, knowledge regarding HIV infection and AIDS was high and consolidated, with almost 100% of correct responses. This is consistent with the results of a study exploring HIV knowledge in a sample of MSM in South Africa and in the United States, which showed a high level of knowledge among MSM living in both countries [24]. However, another study conducted in the UK reported a low level of knowledge among black and minority ethnic MSM [25], and this finding is superimposable to the results of other similar studies conducted in low-income or middle-income countries [26,27]. Reasonably, in high-income countries and among people with a higher social status, HIV knowledge and awareness are adequate, especially in the population at high risk of infection, like MSM. This is coherent with a large body of evidence showing that, if educational level rises, so does HIV knowledge [28,29]. Nevertheless, among MSM, HIV infection has steadily increased over the last few years [30]. Hence, knowledge and awareness do not seem to be sufficient to avoid risky behaviours and prevent HIV infection. Therefore, the implementation of preventive strategies, such as PrEP and condom use, is essential in order to reduce the prevalence of HIV infection among populations at high risk.

In the present sample, more than 90% of the participants were aware of PrEP availability, and almost all of them (87.2%) knew what PrEP is. This is in contrast with the results of an Italian survey conducted among MSM in 2015, which showed that around 25% of the participants have never heard of PrEP [31]. In addition, PrEP awareness in the present sample was higher compared with other international studies in which the proportion of MSM aware of PrEP was, respectively, 64%, 44%, 41% and 54% [12,32,33,34]. A lower PrEP awareness (41.8%) was reported also by a French study conducted among patients with HIV [35]. It thus seems that, in our context, knowledge about PrEP among people at high risk of HIV infection, such as MSM, is satisfactory and that it has significantly increased over the last few years. However, despite a high level of knowledge, in the present study only 7.5% of the respondents declared having used PrEP before. Hence, there is a large discrepancy between PrEP awareness and use, the reasons for which have to be established.

The present work showed that the most significant reasons behind the scarce use of PrEP were: the high cost of the therapy, fear of collateral effects and the feeling of not being a subject at risk of HIV infection.

In Italy, it is possible to access PrEP only with the prescription of a medical doctor specialized in infectious diseases, and the cost of the therapy is not refundable by the National Health System. In order to be safe and effective, PrEP must be taken on a daily basis or according to the “on demand” schedule, which requires 2 doses 24 h before sexual intercourse, followed by a third and a fourth dose after 24 and 48 h respectively. According to the most recent Italian guidelines [3], the “on demand” schedule is admissible only for MSM. This schedule could, therefore, reduce the cost of the therapy for MSM users. However, since in Italy a single box with 30 pills costs around 60 euros, PrEP can easily become greatly expensive even for MSM. This is probably a significant obstacle that limits the diffusion of PrEP use. In the European Union, and subsequently in Italy, PrEP was approved in 2016 [36]. However, in Italy the use of PrEP is still highly unsatisfactory, with an estimated number of people using it of around 400–600 individuals. This number is significantly low, especially if compared to a neighbouring country such as France, which has an estimated number of PrEP users of around 24,000 individuals [37]. This difference is probably due to the fact that in France PrEP has been available since its approval and it is refundable by the National Health System.

In addition, almost 70% of the participants in this survey gained information about PrEP individually on the Internet and around 50% of them throughout friends and acquaintances. Interestingly, almost no one obtained information from the general practitioner, and less than 8% from a specialized physician. These findings are consistent with the results of an Italian study, which showed that, despite 98% of specialized doctors were aware of PrEP, only 14% of them had previously suggested or prescribed PrEP to their patients [15]. Another Italian study showed that, among physicians expert in antiretroviral therapy, almost 50% of them believed that there are insufficient reasons to make PrEP available in Italy [38]. However, PrEP efficacy and safety have been confirmed by a steadily growing body of evidence [5,6,39,40]. In addition, in the present work the majority of the participants stated to be more willing to use PrEP if they could be more informed about it. It is possible that the perception of PrEP as a potentially risky treatment is simply due to a lack of scientific and reliable information coming from health care institutions. Therefore, it is necessary to establish strategies to implement PrEP knowledge among healthcare workers dealing with patients at high risk of HIV infections, enabling them to correctly explain risks and benefits. This could reduce the groundless fear of collateral effects, which undermines the possibility of an effective spreading of PrEP use.

Another interesting result of the present study is that PrEP’s affordability and accessibility determine the will to use it. A survey carried out in England in 2019 showed that 30.9% of users gained PrEP throughout the Internet [41]. This could be due to the fact that, in England, online purchasing of PrEP has been available since 2015 under the supervision of the National Health System. In Italy online purchasing is not possible throughout official institutions and, since in our sample fear of being discriminated against did not emerge as an obstacle, PrEP’s availability and administration should be implemented throughout the official health care services, making online purchasing more difficult to control and regulate.

Furthermore, from the present survey it seems that also LGBT and HIV/AIDS associations do not have a significant role in the diffusion of PrEP knowledge. In fact, in our sample, only 35.6% of the respondents obtained information about PrEP from associations, and attending LGBT and/or HIV/AIDS associations was not a significant factor associated with PrEP knowledge. Another Italian study, exploring PEP (post-exposure prophylaxis) awareness in a sample of MSM, people living with patients with HIV/AIDS and high-risk heterosexuals, showed that the strongest predictor of PEP knowledge was the contact with HIV/AIDS associations, which have a significant role in the diffusion of knowledge and discussion of issues related to HIV/AIDS [42]. This difference could be explained by the fact that, in our sample, the majority of the participants were not enrolled in any HIV/AIDS associations, and therefore being an association member did not emerge as a predictor of knowledge. Other studies are required to assess PrEP awareness among MSM who are actively involved in HIV/AIDS associations, in order to establish if these associations could have an effective role in the spreading of PrEP use.

On the other hand, the present work showed that the most significant factors associated with PrEP knowledge were being single, having had sexual intercourse in the last 12 months with more than one man and regularly undergoing HIV testing. It is likely that single MSM have more sexual intercourse, therefore being at higher risk of contracting HIV. In addition, it is possible that MSM regularly undergoing HIV testing indulge in risky behaviours and hence consider themselves at risk for HIV infection. All together, these results suggest that among MSM with an increased HIV risk the knowledge of PrEP is higher. This is consistent with the fact that, in the present survey, one of the most significant reasons against PrEP use was the feeling of not being a subject at risk of HIV infection. It is likely that only MSM that consider themselves at risk for sexually transmitted diseases are more eager to use PrEP. This could mean that educational and promotional campaigns regarding PrEP specifically targeted on this subgroup of susceptible individuals would probably be highly effective in implementing PrEP use. However, it is also crucial to identify the potential subgroup of subjects who do not consider themselves at risk given that they could miss the opportunity of being informed about PrEP. Furthermore, since HIV-testing seems related to PrEP knowledge, the locations in which HIV-testing takes place, such as pharmacies and clinics, could be exploited for PrEP promotion and distribution, integrating these two key moments in the fight against HIV infection.

### Limitations

The principal limitation of this study is that it was an online cross-sectional survey carried out on a limited sample of 196 MSM. The initial objective was to recruit around 500 subjects from aggregation places targeted on the homosexual population. However, due to the COVID-19 pandemic, we were forced to switch to an online survey, with more difficulty in recruiting eligible participants and possible selection bias. Additionally, questionnaires rely on the honesty of the respondents. However, despite the small number of participants, to our knowledge there are no similar studies conducted in our context on this specific subgroup of population. Hence, the results of this survey, although limited, stimulate interesting thoughts about the implementation of PrEP diffusion and the possible strategies to achieve this important public health goal. Our results can, therefore, be considered a useful starting point for further studies, conducted in different contexts on a larger number of subjects, in order to confirm and strengthen our findings.

## 5. Conclusions

The results of this survey indicate that among MSM knowledge regarding PrEP is well established. However, PrEP use is not equally widespread. The principal obstacles against PrEP use were the high cost of the therapy, the fear of collateral effects and the feeling of not being a subject at risk of HIV infection. In addition, PrEP information did not come from official healthcare workers and institutions. Therefore, it is necessary to implement PrEP knowledge among doctors and other healthcare workers dealing with patients at high risk of HIV infections, enabling them to properly explain risks and benefits. Furthermore, since frequent HIV testing emerged as a strong factor associated with PrEP knowledge, the services for HIV testing and control could be effectively exploited as an occasion for PrEP promotion and distribution.

## Figures and Tables

**Table 1:** Description of the sample (N = 196).

		N	%
Age *		31	10
Nationality	Italian	191	97.4
	Other	5	2.6
Educational level	Middle School	3	1.5
	High School	58	29.6
	University	135	68.9
Profession	Manager	6	3.1
	Worker	8	4.1
	Retired	2	1.0
	Student	35	17.9
	Artisan, shop keeper, businessman	17	8.7
	Health care worker	28	14.3
	Employee	69	35.2
	Unemployed	8	4.1
	Other	23	11.6
Marital status	Single	107	54.6
	In a relation, not cohabitant	48	24.5
	In a relation, cohabitant	28	14.3
	Civilly united/married	13	6.6
Sexual orientation	Exclusively male	146	74.5
	Mainly male	40	20.4
	Both male and female	8	4.1
	Mainly female	2	1.0
Are people close to you aware of your sexual orientation?	No	5	2.6
	Yes, everybody	114	58.2
	Yes, somebody	77	39.2
Do you consider yourself a transgender male?	No	193	98.5
	Yes	3	1.5
Are you enrolled in any LGBT associations?	No	121	61.7
	Yes	75	38.3
Are you enrolled in any associations against AIDS?	No	171	87.2
	Yes	25	12.8
How many men did you have sex with in the last 12 months?	0	13	6.6
	1	46	23.5
	More than 1	137	69.9
Were your partner HIV-positive?	No	110	60.1
	Yes	10	5.5
	I don’t know	63	34.4
Do you have a regular partner?	No	106	54.1
	Yes	90	45.9
Your stable partner is positive for HIV?	No	83	93.3
	Yes	5	5.6
	I don’t know	1	1.1
Do you regularly use condom with your partner?	No	65	73.0
	Yes	24	27.0
Do you use condom during occasional sex?	No	12	6.2
	Yes	182	93.8
Do you know and use STD (sexually transmittable diseases) centres?	I don’t know them	29	14.8
	I know and use them	129	65.8
	I know but do not use them	38	19.4
Did you ever test yourself for HIV?	No	18	9.2
	Yes, once	19	9.7
	Yes, more than once	69	35.5
	Yes, on a regular basis	89	45.6
If yes, when was it the last time?	Less than a year ago	112	63.3
	Between one and two years ago	41	23.2
	Between two and five years ago	16	9.0
	More than five years ago	8	4.5
In the last 12 months, did you have penetrative sex without the use of a condom?	No	85	43.8
	Yes	109	56.2
Have you ever experienced chemsex?	No	161	82.6
	Yes	34	17.4
Have you ever received money in exchange for sex?	No	164	84.5
	Yes	30	15.5
In the last 12 months, have you used intravenous drugs?	No	194	99.5
	Yes	1	0.5
In the last 12 months, have you been rehabilitating from substance abuse?	No	192	98.5
	Yes	3	1.5
In the last 12 months, have you contracted a sexual-transmittable disease?	No	155	79.1
	Yes	34	17.3
	I don’t know	7	3.6
Number of risky behaviours *	Maximum value = 6	2	2

* Value expressed as median and interquartile range.

**Table 2:** HIV knowledge (N = 196).

		N	%
HIV is not transmissible throughout cough or sneeze	False	18	9.2
	True *	177	90.3
	I don’t’ know	1	0.5
HIV and AIDS are the same thing	False *	166	84.7
	True	28	14.3
	I don’t know	2	1.0
It is possible to contract HIV sharing a glass with an infected person	False *	187	95.4
	True	6	3.1
	I don’t’ know	3	1.5
It is possible to contract HIV sharing a syringe with an infected person	False	2	1.0
	True *	194	99.0
	I don’t’ know	0	-
It is possible to contract HIV shaking hands with an infected person	False *	195	99.5
	True	1	0.5
	I don’t’ know	0	-
The onset of symptoms is rapid after HIV infection	False *	186	94.9
	True	0	-
	I don’t’ know	10	5.1
A man can contract HIV having sex with another man	False	6	3.1
	True *	188	95.9
	I don’t’ know	2	1.0
A single sexual intercourse with an infected person is sufficient to contract HIV	False	22	11.2
	True *	166	84.7
	I don’t’ know	8	4.1
It is possible to contract HIV throughout oral sex	False	15	7.7
	True *	168	85.7
	I don’t’ know	13	6.6
Genital washing after sex prevents HIV transmission	False *	173	88.3
	True	7	3.5
	I don’t’ know	16	8.2
Condom use reduce HIV transmission	False	0	-
	True *	196	100
	I don’t’ know	0	-
A person with HIV can appear and feel perfectly healthy	False	1	0.5
	True *	191	97.5
	I don’t’ know	4	2.0
It is possible to be seropositive for many years before developing AIDS	False	1	0.5
	True *	180	91.8
	I don’t’ know	15	7.7
There is a blood test capable of diagnosis HIV infection	False	0	-
	True *	195	99.5
	I don’t’ know	1	0.5
Usually it is possible to recognize a person with HIV simply looking at him	False *	191	97.4
	True	3	1.6
	I don’t’ know	2	1.0
Having sex with multiple partners increases the risk of HIV infection	False	15	7.7
	True *	179	91.3
	I don’t’ know	2	1.0
Having already contracted a sexual-transmittable disease increases the risk of HIV infection	False	57	29.1
	True *	98	50.0
	I don’t’ know	41	20.9
There is a vaccine for the prevention of HIV infection	False *	176	89.8
	True	11	5.6
	I don’t’ know	9	4.5
Correct answers ^§^		17	1
Percentage of correct answers ^§^		94.4	5.6

* Correct answer. ^§^ Value expressed as median and interquartile range.

**Table 3:** Knowledge, attitudes and practices regarding pre-exposure prophylaxis (PrEP).

**Principal Outcomes**			
		**N**	**%**
Have you ever heard of PrEP before?	*No*	17	8.9
	*Yes*	174	91.1
If yes, do you know what PrEP is?	*No*	22	12.8
	*Yes*	150	87.2
If yes, have you ever used PrEP?	*No*	160	92.5
	*Yes*	13	7.5
Have you ever talked about PrEP with friends or relatives?	*No*	55	31.6
	*Yes*	119	68.4
Have you ever talked about PrEP with a health care worker?	*No*	114	65.5
	*Yes*	60	34.5
Do you have any friends, relatives or acquaintances using PrEP?	*No*	94	49.2
	*Yes*	97	50.8
Would you be more willing to use PrEP if you had more information?	*No*	52	27.4
	*Yes*	99	52.1
	*I don’t know*	39	20.5
Would you be more willing to use PrEP if it were free?	*No*	35	18.3
	*Yes*	127	66.5
	*I don’t know*	29	15.2
Would you be more willing to use PrEP if it were available in pharmacy without medical prescription?	*No*	48	25.3
	*Yes*	109	57.4
	*I don’t know*	33	17.4
**Sources of PrEP information**			
		**N**	**%**
Internet	*No*	55	31.6
	*Yes*	119	68.4
TV	*No*	166	95.4
	*Yes*	8	4.6
Informative brochures	*No*	134	77.0
	*Yes*	40	23.0
Institutional channels (School, University, Ministry of Health, …)	*No*	156	89.7
	*Yes*	18	10.3
Associations	*No*	112	64.4
	*Yes*	62	35.6
Friends, relatives, acquaintances	*No*	91	52.3
	*Yes*	83	47.7
Partner	*No*	156	89.7
	*Yes*	18	10.3
General practitioner	*No*	173	99.4
	*Yes*	1	0.6
Specialist physician	*No*	161	92.5
	*Yes*	13	7.5
Other	*No*	191	97.4
	*Yes*	5	2.6
**Reasons for not using PrEP**			
		**N**	**%**
Too expensive	*No*	117	73.1
	*Yes*	43	26.9
Difficult to purchase	*No*	133	83.1
	*Yes*	43	26.9
Fear of being discriminated	*No*	154	96.3
	*Yes*	6	3.8
Fear of collateral effects	*No*	122	76.3
	*Yes*	38	23.8
I think it is not effective	*No*	144	90.0
	*Yes*	16	10.0
I’m not a subject at risk	*No*	126	78.8
	*Yes*	34	21.3
Other	*No*	133	67.9
	*Yes*	63	32.1

**Table 4:** Univariate analysis for PrEP knowledge.

		PrEP Knowledge		
		No N (%)	Yes N (%)	*p*-Value
Educational level	High school (or lower)	10 (19.2)	42 (80.8)	0.096
	Other	12 (10.0)	108 (90.0)	
Single	No	15 (19.2)	63 (80.8)	0.021
	Yes	7 (7.4)	87 (92.6)	
Sexual orientation	Males only	17 (13.1)	113 (86.9)	0.843
	Mostly males/Both males and females/Mostly females	5 (11.9)	37 (88.1)	
Are people close to you aware of your sexual orientation?	No/Somebody	8 (11.8)	60 (88.2)	0.745
	Yes, everybody	14 (13.5)	90 (86.5)	
Do you consider yourself a transgender male?	No	21 (12.4)	149 (87.6)	0.113
	Yes	1 (50.0)	1 (50.0)	
Are you a member of a LGBT association?	No	17 (16.7)	85 (83.3)	0.066
	Yes	5 (7.1)	65 (92.9)	
Are you a member of an association fighting AIDS?	No	22 (14.8)	127 (85.2)	0.048
	Yes	0 (0)	23 (100)	
How many men did you have sex with in the last 12 months?	0/1	12 (25.5)	35 (74.5)	0.002
	More than 1	10 (8.0)	115 (92.0)	
Were your partners HIV-positive?	No	17 (18.1)	77 (81.9)	0.026
	Yes	0 (0)	9 (100)	
	I don’t know	3 (4.9)	58 (95.1)	
In the last 12 months, did you have penetrative sex without the use of a condom?	No	10 (14.5)	59 (85.5)	0.483
	Yes	11 (10.9)	90 (89.1)	
Do you know and use STD (sexually transmittable diseases) centres?	No	11 (22.0)	39 (78.0)	0.021
	Yes	11 (9.0)	111 (91.0)	
Do you regularly test yourself for HIV?	No	16 (18.8)	69 (81.2)	0.021
	Yes	6 (7.0)	80 (93.0)	
Have you ever experienced chemsex?	No	19 (13.9)	118 (86.1)	0.204
	Yes	2 (5.9)	32 (94.1)	
Have you ever received money in exchange for sex?	No	21 (14.6)	123 (85.4)	0.121
	Yes	1 (3.7)	26 (96.3)	
In the last 12 months, have you contracted a sexual-transmittable disease?	No	16 (12.0)	117 (88.0)	0.581
	Yes	6 (15.4)	33 (84.6)	

**Table 5:** Multivariate analysis for PrEP knowledge.

		PrEP Knowledge	
		OR (CI95%)	AdjOR (CI95%)
Educational level	High School (or lower)	-	-
	Other	2.14 (0.86–5.33)	3.92 (1.36–11.38)
Single	No	-	-
	Yes	2.96 (1.14–6.01)	2.543 (0.88–7.35)
How many men did you have sex with in the last 12 months?	0/1	-	-
	More than 1	3.94 (1.48–6.89)	2.70 (0.95–7.64)
Do you regularly test yourself for HIV?	No	-	-
	Yes	3.09 (1.15–8.34)	3.16 (1.06–9.29)
Have you ever received money in exchange for sex?	No	-	-
	Yes	4.44 (0.57–34.49)	4.58 (0.56–37.78)
